# The cost effectiveness of toripalimab plus bevacizumab versus sorafenib for the first-line treatment of advanced hepatocellular carcinoma in China

**DOI:** 10.3389/fphar.2026.1726444

**Published:** 2026-03-16

**Authors:** Ling Ma, Ting Zhu, Jihong Duan, Niya Huang, Zhiqing Zhang

**Affiliations:** 1 Department of Clinical pharmacy, the First People’s Hospital of Yunnan Province, the Affiliated Hospital of Kunming University of Science and Technology, Kunming, Yunnan Province, China; 2 Mile People’s Hospital, Mile, Yunnan Province, China; 3 Jinghong First People’s Hospital, Xishuangbanna Dai Autonomous Prefecture, Yunnan Province, China

**Keywords:** China, cost-effectiveness, hepatocellular carcinoma, partitioned survival approach, sorafenib, toripalimab

## Abstract

**Background:**

Supported by evidence of efficacy and safety from the landmark HEPATORCH trial, the combination therapy of toripalimab and bevacizumab (TORI + Bev) has been adopted into the Chinese Society of Clinical Oncology (CSCO) clinical guidelines for advanced hepatocellular carcinoma (HCC). The economic viability of this regimen, however, has yet to be assessed.

**Methods:**

This economic evaluation used a partitioned survival model (PartSA) over a 10-year horizon. Overall survival data from the HEPATORCH trial were incorporated into the model to compare the cost-effectiveness of first-line TORI plus bevacizumab (TORI + Bev) *versus* sorafenib in advanced hepatocellular carcinoma (HCC). Cost inputs were limited to direct medical expenditures: drug prices were obtained from the National Drug Price Database of Yaozh.com, and other cost and utility parameters were sourced from published literature. Considering the unique characteristics of China’s centralized drug procurement policy and the substantial price differences between originator and generic drugs within the same therapeutic class, this study stratify analysis by originator vs. generic scenarios separately. The primary outcome was the incremental cost-effectiveness ratio (ICER). Sensitivity analyses were performed to evaluate the robustness of the model results.

**Results:**

Compared with sorafenib, the TORI plus bevacizumab regimen demonstrated greater health benefits (1.62 QALYs vs. 1.27 QALYs). However, due to its higher treatment costs, the incremental cost-effectiveness ratio (ICER) for TORI plus bevacizumab reached $67,352.09 per QALY under originator pricing and $65,300.63 per QALY under generic pricing. Probabilistic sensitivity analysis indicated that, when using three times China’s GDP *per capita* ($40,723.4) as the cost-effectiveness threshold, the probability of TORI plus bevacizumab being cost-effective was 9.2% for originator pricing and 9.1% for generic pricing.

**Conclusion:**

TORI + Bev is unlikely to be cost effective compared with sorafenib for the first-line treatment of advanced HCC in China. Reducing the price of bevacizumab can increase the possibility of it being cost effective in the future.

## 1 Introduction

Primary liver cancer ranks as the sixth most commonly diagnosed malignancy and the third leading cause of cancer-related mortality worldwide, with China bearing nearly half of the global disease burden (Zhou et al., 2023; Bray et al., 2024). Hepatocellular carcinoma (HCC) accounts for approximately 90% of primary liver cancer cases (Zhou et al., 2023; Llovet et al., 2021; Siegel et al., 2023). Infection is a key risk factor for HCC, with hepatitis B virus (HBV) infection contributing to 50%–80% of cases worldwide. China, as a country with a high burden of HBV infection, exhibits a particularly prominent proportion of HCC cases driven by HBV infection (Yan et al., 2025). Clinically, a large proportion of HCC patients are diagnosed at advanced stages, resulting in a 5-year relative survival rate below 20% (Llovet et al., 2024; Chidambaranathan-Reghupaty et al., 2021). Furthermore, although most patients present with initially unresectable tumors, even among those who undergo radical resection, over 70% experience disease progression or recurrence within 5 years after surgery (Llovet et al., 2024; Yao et al., 2022; Galle et al., 2021).

The HEPATORCH trial (NCT04723004) was a randomized, open-label, phase III study conducted across 57 hospitals in mainland China, Taiwan, and Singapore, enrolling patients with hepatocellular carcinoma, among whom the hepatitis B virus infection rate was 89.9% (Shi et al., 2025). The findings indicated that toripalimab plus bevacizumab (TORI + Bev) brought significant prolongations in both overall survival (OS) and progression-free survival (PFS). Comparing with sorafenib, the median OS was 20.0 vs. 14.5 months (hazard ratio (HR) = 0.76, 95% confidence interval (CI): 0.58–0.99), the median PFS was 5.8 vs. 4.0 months (HR = 0.69, 95% CI: 0.53–0.91). Based on the positive results, TORI + Bev has been recommended as a new first-line treatment for advanced HCC in the 2025 edition of Chinese Society of Clinical Oncology (CSCO) guidelines.

Although the combination of immune checkpoint inhibitors (ICIs) and bevacizumab has demonstrated established clinical efficacy and safety for the treatment of advanced HCC, it is crucial to evaluate its cost-effectiveness, as the high treatment costs may impose a significant economic burden on the healthcare system. However, current pharmacoeconomic evaluations have primarily focused on agents such as sintilimab, pembrolizumab, and atezolizumab, leaving the cost-effectiveness of toripalimab-based regimens underexplored (Xu et al., 2023; Zhou et al., 2022a, 2022b; Gong et al., 2023; Wen et al., 2024). In this study, we evaluated the cost-effectiveness of TORI + Bev *versus* sorafenib as a first-line treatment for advanced HCC from the perspective of the Chinese healthcare system.

## 2 Methods

The patient characteristics and interventions used in this model were based on the HEPATORCH trial (Shi et al., 2025). As no human subjects were directly involved in this modeling study, institutional review board (IRB) review or ethics committee exemption was not required.

The HEPATORCH trial enrolled 326 patients with advanced hepatocellular carcinoma (HCC). Key inclusion criteria were: age 18–75 years; Child-Pugh class A liver function without a history of hepatic encephalopathy; baseline Eastern Cooperative Oncology Group (ECOG) performance status of 0 or 1; and adequate organ function. Exclusion criteria included prior gastrointestinal bleeding within the past 6 months; significant coagulation disorders or other clear evidence of bleeding tendency; and active coinfection with hepatitis B virus (HBV) and hepatitis C virus (HCV). Patients were randomized to receive either TORI + Bev (n = 162) or sorafenib monotherapy (n = 164). The intervention group received intravenous toripalimab 240 mg plus bevacizumab 15 mg/kg body weight every 3 weeks, while the control group received oral sorafenib 400 mg twice dail. This treatment regimen ensured comparability between groups. Therapy was continued until disease progression, intolerable toxicity, withdrawal of informed consent, or completion of 35 cycles of toripalimab. Patients who discontinued first-line treatment received subsequent therapies until death. The model incorporated demographic parameters representative of the Chinese population, including an average body weight of 64.8 kg (National Health and Family Planning Commission.2020).

Given that the HEPATORCH trial reported only the distribution of second-line systemic therapies—including chemotherapy, targeted therapy, radiotherapy, and immunotherapy—administered following disease progression on initial treatment, and that targeted therapy represented the most common category without further specification of the precise regimens used, we derived second-line treatment assumptions based on the Chinese Society of Clinical Oncology (CSCO) Guidelines for the Diagnosis and Treatment of Liver Cancer (2025). In accordance with these guidelines, we assumed that regorafenib or sorafenib would be used as second-line treatments for patients initially treated with the “Toripalimab plus Bevacizumab” regimen. Similarly, we assumed that regorafenib or apatinib would be administered as second-line therapies for patients whose first-line treatment was sorafenib. We adopted this guideline-based approach to reflect real-world clinical practice in China and ensure consistency across treatment arms. While this introduces some structural uncertainty (e.g., regarding the actual distribution, costs, and effectiveness of second-line regimens), it is a necessary step to minimize potential bias in our comparison.

### 2.1 Model structure

**FIGURE 1:**
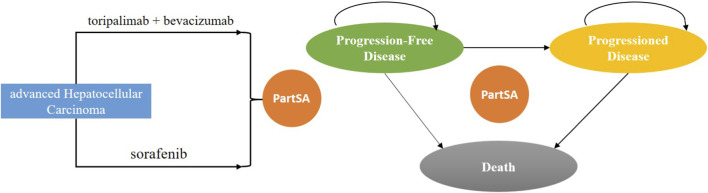
Diagram of the model structure.

A partitioned survival model (PSM) was developed in R software (version 4.3.3) to evaluate the long-term costs and health outcomes associated with TORI + Bev *versus* sorafenib in the treatment of advanced HCC. The PSM estimates the proportion of patients in each health state using a set of independently modeled, non-mutually exclusive survival curves, a methodology widely employed in the economic evaluation of anticancer therapies (Bullement et al., 2019). The model comprises three health states: progression-free (PF), progressive disease (PD), and death. All patients entered the model in the PF state. From there, individuals could remain progression-free, transition to the PD state, or die. Those in the PD state could either remain in that state or die. The model structure is illustrated in Figure 1. The cycle length in the model was based on the treatment schedule, with toripalimab plus bevacizumab administered on a 21-day cycle and targeted therapy on a 30-day cycle. The time horizon was set as 10 years, with 99% of people dying. This analysis was performed from the perspective of Chinese healthcare system.

**FIGURE 2:**
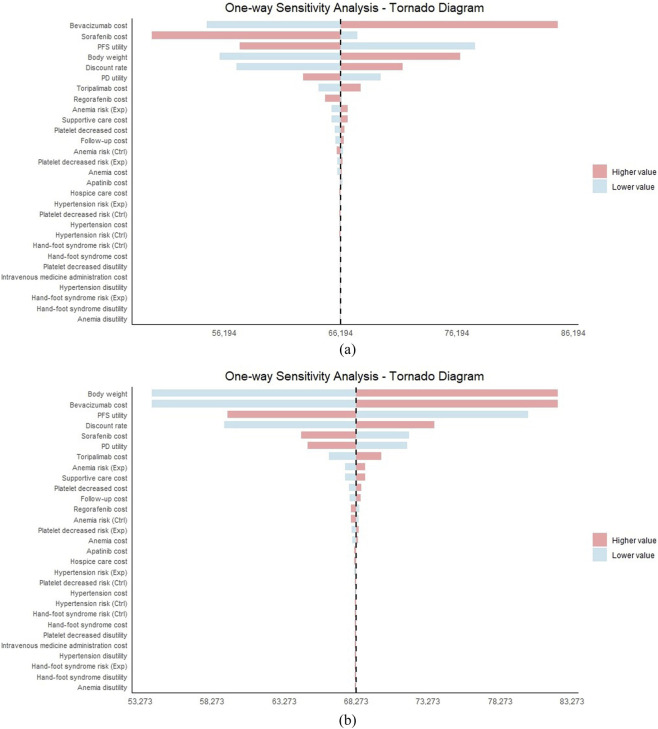
One-way sensitivity analysis. **(a)** Based on originator drug prices. **(b)** Based on generic drug prices.

WebPlotDigitizer was used to extract time-to-survival data from survival curves. Subsequently, R language software (version 4.3.3) was employed to reconstruct individual time-to-event data and extrapolate survival curves based on Guyot et al.‘s algorithm (Guyot et al., 2012). Various parametric survival models, including Exponential, Weibull, Log-normal, Log-logistic, Gompertz, and Gamma, were utilized for curve fitting and extrapolation. The parametric models that had the smallest Akaike Information Criterion (AIC) and Bayesian Information Criterion (BIC) values were determined to be the best-fitted models (Supplementary Table S1; Supplementary Figures S1). Distributions and key parameters of the optimal survival curves are summarized in Table 1. However, it is important to acknowledge that survival extrapolation beyond the observed trial period introduces uncertainty, as long-term survival outcomes may deviate from model predictions Figure 2.

**TABLE 1:** Parameters of the best-fitted distributions.

Kaplan meier survival curve	Best fitted distribution	Key parameters
OS curve of TORI + Bev arm	Log-normal	Meanlog = 2.9478, sdlog = 1.1171
OS curve of sorafenib arm	Log-normal	Meanlog = 2.6978, sdlog = 1.0499
PFS curve of TORI + Bev arm	Log-normal	Meanlog = 1.7326, sdlog = 1.1048
PFS curve of sorafenib arm	Log-normal	Meanlog = 1.3426, sdlog = 0.9003

OS, overall survival; PFS, progression-free survival; PD, progressive disease.

### 2.2 The utilities

Survival time in each disease state was weighted by utility scores and converted into quality-adjusted life years (QALYs) for each treatment. Health-related quality of life, measured using the EQ-5D instrument, was extracted to assess cost-effectiveness across groups (Rabin and de Charro, 2001). Health state utility values and disutility values due to serious adverse events were derived from previously published literature (Péter et al., 2014; Wen et al., 2024). Detailed values are presented in Table 2.

**TABLE 2:** Key model inputs.

Model inputs	Base-case value (range)	distribution	Source
Cost	​	​	​
Routine follow-up cost per cycle	114 (91.2–136.8)	Gamma	Li and Wan (2023)
Supportive care per cycle	210 (168–252)	Gamma	Li and Wan (2023)
Intravenous medicine administration	2.66 (2.13–3.19)	Gamma	Shen et al. (2022)
Terminal care	1839 (1471.2–2206.8)	Gamma	Li and Wan (2023)
Patient weight(kg)	64.8 (51.84–77.76)	Normal	National Health and Family Planning Commission (2020)
Discount rate(%)	5 (0–8)	Normal	Li and Wan (2023)
Cost of drug	​	​	​
Toripalimab/240 mg	267.36 (213.88–320.83)	Gamma	​
Bevacizumab/100 mg	Originator 212.77 (170.22–255.32) Generic 156.14 (124.91–187.37)	Gamma	​
Sorafenib/200 mg	Originator 12.67 (10.14–15.20) Generic 1.75 (0.78–2.81)	Gamma	​
Regorafenib/40 mg	Originator 17.13 (13.701–20.56) Generic 3.03 (0.60–16.67)	Gamma	​
Apatinib/250 mg	14.85 (11.88–17.82)	Gamma	​
Cost of serious adverse events	​	​	​
Thrombocytopenia	2072.18 (1657.74–2486.62)	Gamma	Gao et al. (2024)
Hypertension	44.88 (35.90–53.86)	Gamma	Gao et al. (2024)
Palmar–plantar erythrodysesthesia syndrome per event	10.45 (8.36–12.54)	Gamma	Liao et al. (2024)
Anaemia	417.15 (333.72–500.58)	Gamma	1
Utility	​	​	​
PFS	0.76 (0.608–0.912)	Beta	Wen et al. (2024)
PD	0.68 (0.544–0.816)	Beta	Wen et al. (2024)
Disutility of treatment-emergent adverse event	​	​	​
Thrombocytopenia	0.05 (0.088–0.132)	Beta	Gao et al. (2024)
Hypertension	0.04 (0.048–0.032)	Beta	Gao et al. (2024)
Anaemia	0.07 (0.056–0.084)	​	​
Palmar–plantar erythrodysesthesia syndrome	0.116 (0.093–0.139)	​	Meng et al. (2022)
Risk of serious adverse events in TORI + Bev group (%)	​	​	​
Thrombocytopenia	10 (8–12)	Beta	Shi et al. (2025)
Anaemia	6 (4.8–7.2)	Beta	Shi et al. (2025)
Hypertension	16 (12.8–19.2)	​	Shi et al. (2025)
Palmar–plantar erythrodysesthesia syndrome	0	​	Shi et al. (2025)
Risk of serious adverse events in sorafenib group (%)	​	​	​
Thrombocytopenia	2 (1.6–2.4)	Beta	Shi et al. (2025)
Anaemia	4 (3.2–4.8)	Beta	Shi et al. (2025)
Hypertension	12 (9.6–14.4)	Beta	Shi et al. (2025)
Palmar–plantar erythrodysesthesia syndrome	10 (8–12)	Beta	Shi et al. (2025)

OS, overall survival; PFS, progression-free survival; PD, progressive disease; AE, adverse event.

### 2.3 Measurement of costs

Our analysis was limited to direct medical costs, which included drug costs, intravenous administration, routine follow-ups, imaging examinations, end-of-life care, and the management of severe adverse events. After consultation with clinical experts, we determined that the monitoring strategies for the two treatment regimens were largely similar. Therefore, the costs of routine check-ups and imaging procedures were assumed to be identical on a per-cycle basis for both groups. Drug prices were obtained from the National Drug Price Database of Yaozh.com (www.yaozh.com). representing typical pricing in most Chinese public hospitals. Dosing was based on an average body weight of 64.8 kg (National Health and Family Planning Commission.2020), and dose wastage for bevacizumab was accounted for, as it is only available in a single specification of 4 mL:100 mg. Other cost inputs were derived from previously published studies (Liao et al., 2024; Gao et al., 2024; Li and Wan, 2023). Patients were assumed to initiate second-line treatment upon disease progression. Since grade 1–2 adverse events are generally manageable, only those of grade 3 or higher with an incidence greater than 5% were included in the analysis. All costs were inflated to 2023 values using the Consumer Price Index and converted to U.S. dollars at an exchange rate of USD 1 = CNY 7.0467. In accordance with the 2020 Chinese Guidelines for Pharma coeconomic Evaluations (CGPE) (Liu et al., 2020), both costs and quality-adjusted life-years (QALYs) were discounted at an annual rate of 5%(0%–8%), and a half-cycle correction was applied to the outcomes.

### 2.4 Sensitivity analysis

We conducted both one-way deterministic and probabilistic sensitivity analyses to evaluate the robustness of the model outcomes. The one-way analysis assessed the impact of varying individual parameters on the results. The unit costs of pharmaceuticals in this study were sourced from the National Drug Price Database of Yaozh.com (www.yaozh.com). This platform systematically collects actual procurement price information from provincial drug centralized purchasing platforms and public healthcare institutions across China, reflecting current real-world price levels in the Chinese pharmaceutical market. In this study, “originator” refers to the innovator brand-name drug that was first authorized for marketing (usually initially protected by patents), as opposed to generic versions that enter the market after patent expiration. Considering the unique characteristics of China’s centralized drug procurement policy and the substantial price differences between originator and generic drugs within the same therapeutic class, this study stratify analysis by originator vs. generic scenarios separately. Consistent with the 2020 CGPE, originator drug prices were anchored to their market prices, and the corresponding parameter range was defined as the baseline value ±20%. The price of generic drugs was calculated as the weighted average based on national bid-winning prices, usage patterns, and procurement policies. For parameter range setting, if the fluctuation between the lowest and highest bid/hanging prices of generic drugs exceeded ±20% of their mean price, the actual lowest and highest prices were used as the parameter range for sensitivity analysis. If the fluctuation was less than ±20% of the mean, a uniform range of ±20% around the mean was applied as the parameter range. Ranges for other parameters were set at ±20% of their base-case values. The results of 1,000 Monte Carlo simulations for estimating ICERs are presented in the cost-effectiveness acceptability curve. Cost parameters and body weight were modeled using gamma distributions, while utility values and adverse event incidence rates followed beta distributions (Table 2).

## 3 Result

### 3.1 Base-case results

Patients treated with TORI + Bev achieved 1.62 QALYs, while those receiving sorafenib attained 1.27 QALYs (Table 3). Under originator drug pricing, total costs were $40,146.70 for TORI + Bev and $16,573.47 for sorafenib. When generic drug pricing was applied, costs decreased to $33,426.20 and $10,570.98, respectively. The incremental cost-effectiveness ratio (ICER) reached $67352.09/QALY with originator pricing and $65,300.63/QALY with generic pricing, both exceeding the Chinese willingness-to-pay (WTP) threshold of $40,723.40 per QALY. Thus, from a Chinese health-economic perspective, the TORI + Bev regimen is not cost-effective as a first-line systemic therapy for advanced HCC. The probability of TORI + Bev being cost-effective varied substantially across WTP thresholds. The threshold required to achieve a 50% probability of cost-effectiveness aligned with the ICER: $67,622.38 per QALY under originator pricing and $65,454.55 per QALY under generic pricing. To attain a high probability of cost-effectiveness (approximately 95%), considerably higher WTP thresholds were needed—$183,000.00 per QALY in the originator scenario and $173,000.00 per QALY in the generic scenario. These findings indicate that while pricing choices modestly influence threshold values, TORI + Bev is unlikely to be cost-effective within China’s currently accepted WTP limits.

**TABLE 3:** Results of the base-case, subgroup, and scenario analyses.

Group	Total cost ($)	Incremental costs ($)	Overall QALYs	Incremental QALYs	ICER ($/QALYs)
Originator pricing	​	​	​	​	​
TORI + Bev	40,146.70	23,573.23	1.62	0.35	67,352.09
Sorafenib	16,573.47	​	1.27	​	​
Generic pricing	​	​	​	​	​
TORI + Bev	33,426.20	22,855.22	1.62	0.35	65,300.63
Sorafenib	10,570.98	​	1.27	​	​

QALYs, quality-adjusted life-years; ICER, incremental cost-effectiveness ratio.

### 3.2 Sensitivity analysis

A one-way sensitivity analysis was performed to evaluate the responsiveness and robustness of the model, with results summarized in a tornado diagram (Figure 2).

Under generic drug pricing, the price of bevacizumab showed the strongest influence on the results, followed by the price of sorafenib and the utility value associated with progression-free survival (PFS). Under originator drug pricing, body weight and the price of bevacizumab together had the greatest impact, closely followed by the PFS utility value and the discount rate.

**FIGURE 3:**
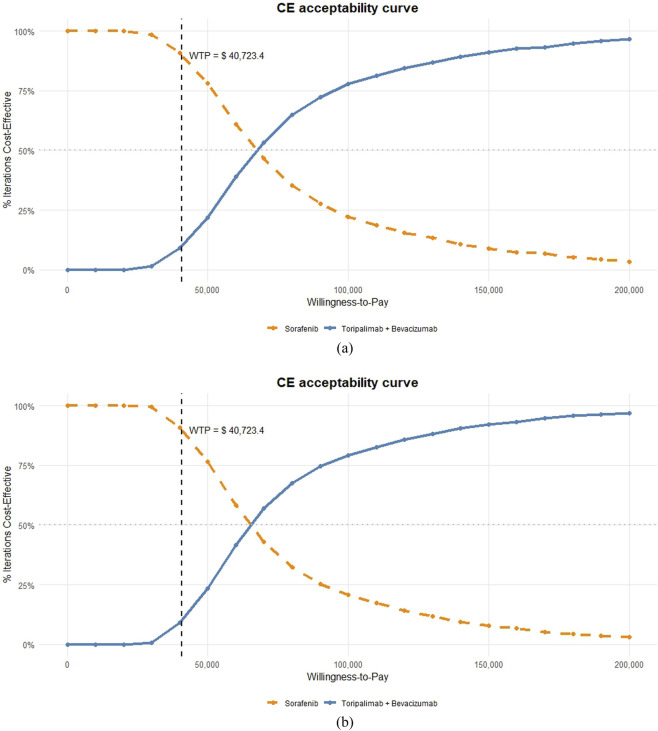
Cost-effectiveness acceptability curve.

**FIGURE 4:**
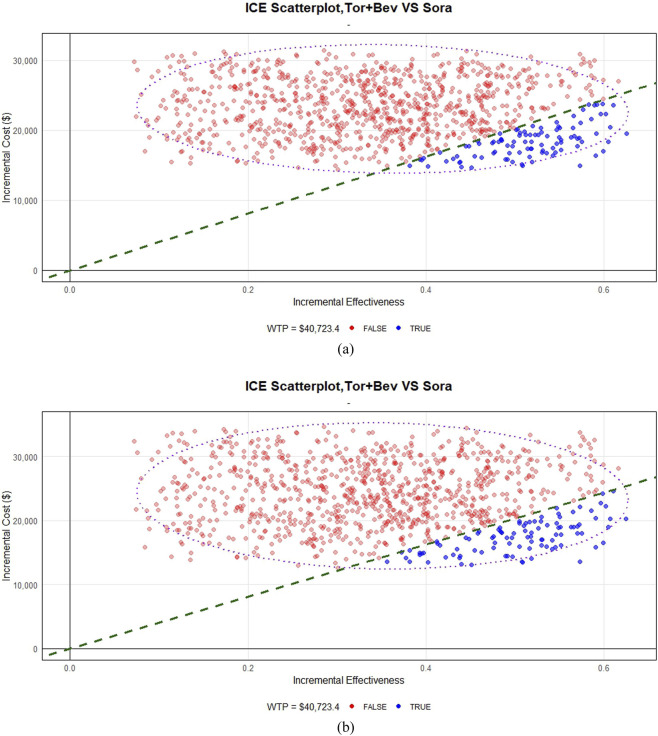
Probabilistic sensitivity analysis scatter plot. **(a)** Based on originator drug prices. **(b)** Based on generic drug prices.

The cost-effectiveness acceptability curve indicated similarly low probabilities of cost-effectiveness for TORI + Bev under both pricing scenarios at China’s current willingness-to-pay (WTP) threshold (9.2% for originator vs. 9.1% for generic) (Figures 3,4).

## 4 Discussion

This study develops an economic model to evaluate TORI + Bev for advanced hepatocellular carcinoma within the clinical context of China. The model is built upon data from the HEPATORCH trial, which provides a highly representative patient cohort with characteristics specific to the Chinese population, including an 89.9% hepatitis B virus infection rate. Although the TORI + Bev regimen demonstrates survival advantages over sorafenib, the analysis finds that its ICER exceeds China’s WTP threshold Figure 4.

Our analysis reveals that the model is highly sensitive to the price of bevacizumab, a feature with important policy implications. Under the current pricing mechanism, combining anti-vascular endothelial growth factor agents with immune checkpoint inhibitors significantly increases treatment costs. At the same time, this suggests that reducing drug prices—particularly for bevacizumab—through mechanisms such as government negotiation could fundamentally improve the cost-effectiveness of treatment regimens.

Economic evaluations based on localized data not only inform decision-making for specific treatment options but also provide critical insights for healthcare resource allocation. This study recommends that, under constrained budgets, policymakers should prioritize more cost-effective intervention strategies (such as regular screening for patients with cirrhosis) to maximize population health benefits through optimized resource allocation. This research paradigm and its findings hold significant value for establishing a precision decision-making system aligned with China’s disease profile and healthcare economic conditions.

To our knowledge, this is the first cost-effectiveness analysis of toripalimab plus bevacizumab *versus* sorafenib for advanced HCC conducted specifically from a Chinese payer perspective. While most existing economic evaluations of HCC immunotherapies derive from U.S. studies, their conclusions vary significantly. For instance, although atezolizumab plus bevacizumab demonstrates superior efficacy and safety over sorafenib in the U.S. setting, its incremental cost-effectiveness ratio exceeds conventional thresholds, rendering it not cost-effective (Zhang et al., 2021). Similarly, despite proven clinical advantages *versus* sorafenib, nivolumab falls outside acceptable ICER ranges in U.S. economic assessments (Li et al., 2022). Not all immunotherapies follow this pattern, however: under the U.S. Medicare framework, durvalumab plus tremelimumab demonstrates clear cost-saving advantages relative to sorafenib (Liao et al., 2024). These cost-effectiveness differences are likely driven by three key factors: drug pricing, the modeled durability of survival benefit, and willingness-to-pay (WTP) thresholds. Although toripalimab benefits from competitive pricing within China’s healthcare system, our analysis shows that the TORI + Bev regimen does not meet China’s current WTP threshold of $40,723 per QALY, with a cost-effectiveness probability below 10% at this threshold. These findings highlight several important considerations. First, while drug pricing strategies substantially influence cost-effectiveness outcomes, they interact with other critical variables—including the magnitude of clinical benefit, the durability of survival gains, and adverse event management costs. Second, even under generic pricing, the ICER for TORI + Bev ($65,301/QALY) remains substantially above China’s WTP threshold, indicating that further price reductions or evidence of enhanced long-term survival benefits would be necessary for this regimen to achieve cost-effectiveness within current parameters. Third, the observation that a regimen can be more favorably priced than its U.S.-evaluated counterparts yet still fail cost-effectiveness criteria underscores the relatively stringent nature of China’s WTP threshold and the importance of maintaining realistic expectations regarding immunotherapy value in the Chinese context. More broadly, these contrasting results reveal a pivotal methodological insight: economic evaluations are highly sensitive to local contexts. Accordingly, healthcare decision-makers should exercise caution when extrapolating findings from foreign analyses. Together, these findings reinforce the necessity of rigorous, context-specific economic evaluations to inform reimbursement decisions—rather than assuming that competitive pricing alone ensures cost-effectiveness—and emphasize the need for homegrown economic evidence to guide China’s reimbursement policies and clinical guidelines for advanced HCC.

This study also has several limitations. First, the economic analysis does not compare the regimen with other ICIs-based combinations that have demonstrated clinical benefits, such as atezolizumab-bevacizumab, camrelizumab-rivoceranib, and sintilimab-bevacizumab. Due to the current lack of head-to-head clinical trial data, a direct comparison is not feasible. However, as more evidence becomes available, future research should consider indirect economic evaluations using network meta-analysis to inform comparative cost-effectiveness. Second, the extrapolation of survival curves introduces uncertainty, which may affect the precision of the results. Although no universally accepted method currently exists to fully address this challenge, validation using real-world evidence and extended follow-up studies is warranted. Third, the reliance on utility values from previous literature, rather than values directly from the HEPATORCH trial, represents a potential limitation regarding the generalizability of our quality-of-life adjustments. In defense of our approach, however, subsequent one-way sensitivity analyses revealed that the model’s cost-effectiveness conclusions were insensitive to changes in these utility estimates, suggesting that any such discrepancy is unlikely to affect our primary findings.

In conclusion, the combination of toripalimab and bevacizumab provides a new option for advanced HCC patients. However, the findings of this economic evaluation indicate that, at the current price, it is unlikely to be a cost-effective first-line treatment compared to sorafenib in the Chinese healthcare setting. A reduction in price or optimization the dosage of treatment will increase the probability of it being cost effective.

## Data Availability

The original contributions presented in the study are included in the article/Supplementary Material, further inquiries can be directed to the corresponding author.
