# Supplementary material for: The cost effectiveness of toripalimab plus bevacizumab versus sorafenib for the first-line treatment of advanced hepatocellular carcinoma in China
Source: Front Pharmacol. 2026 Mar 16;17:1726444. doi: 10.3389/fphar.2026.1726444 (PMC13033761; doi:10.3389/fphar.2026.1726444)
Supplement: Supplementary file 1 [file DataSheet1.pdf]

Supplementary Material

Supplementary Table 1. Summary of the statistical goodness-of-fit of Kaplan Meier survival curves.

PFS survival curve				
	Toripalimab,AIC	Toripalimab,BIC	placebo,AIC	placebo,BIC
Exponential	648.262	641.1744	619.0869	611.987
Weibull	647.4472	633.272	613.2883	599.0886
Gamma	645.9602	631.785	607.3371	593.1374
Gompertz	649.7758	639.995	621.0715	606.8718
Log-normal	635.4535	625.6727	586.7854	572.5857
Log-logistic	643.1262	633.3455	591.4485	577.2488
OS survival curve				
	Toripalimab,AIC	Toripalimab,BIC	placebo,AIC	placebo,BIC
Exponential	903.0152	895.9276	1028.249	1021.1493
Weibull	902.5183	888.3431	1024.574	1010.3746
Gamma	900.8154	886.6402	1022.592	1008.3923
Gompertz	905.011	895.2303	1028.956	1014.7567
Log-normal	888.4886	878.7079	1016.599	1002.3989
Log-logistic	895.3354	885.5547	1019.231	1005.0309

PFS, progression-free survival; OS, overall survival; AIC, Akaike's information criterion; BIC, Bayesian information criterion.

(a)

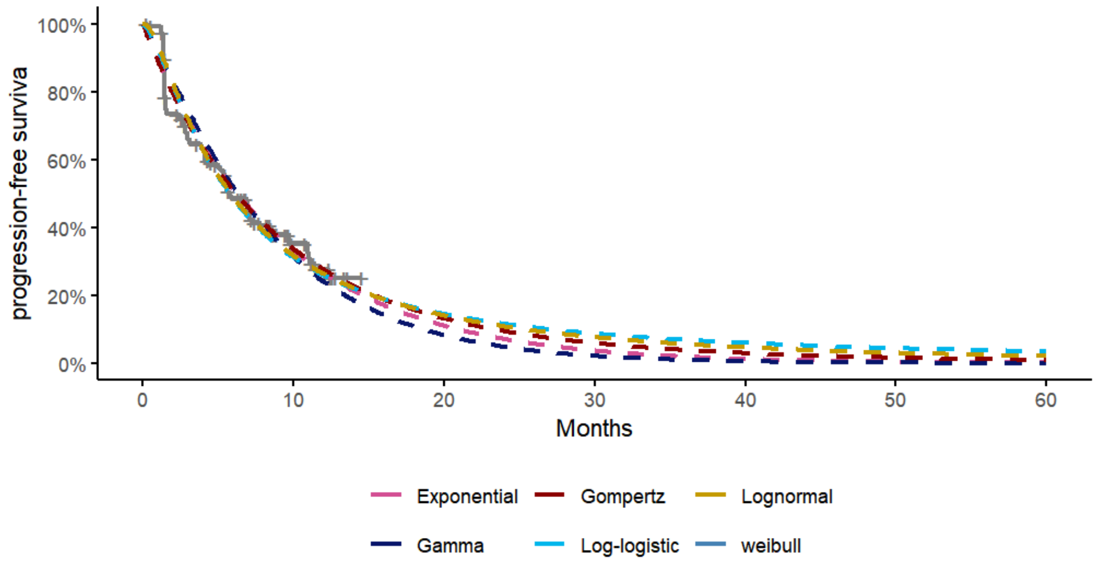

(b)

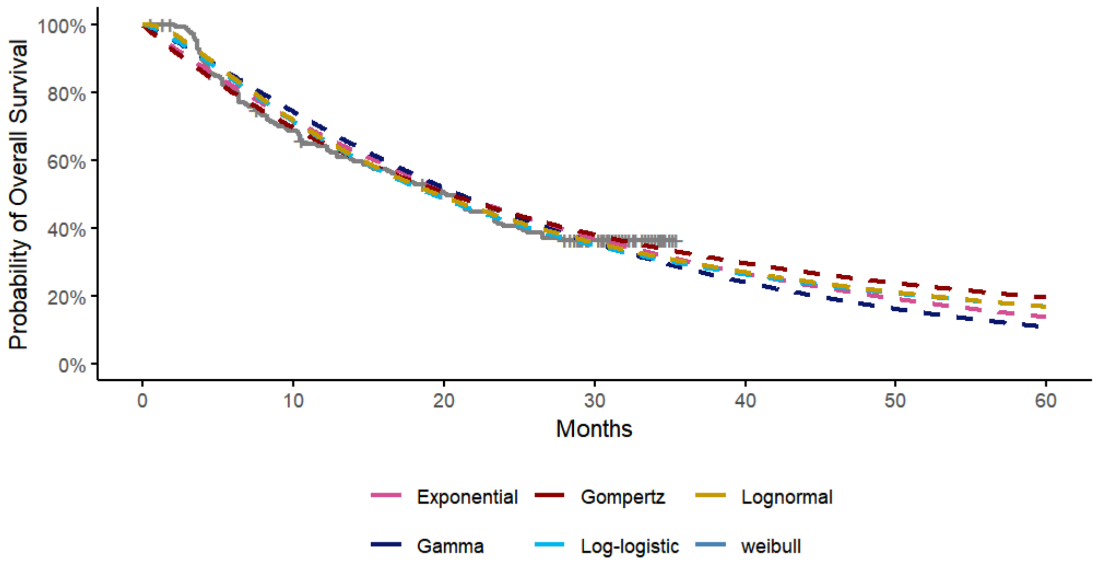

Supplementary Figure 1. Fitting and extrapolation of Kaplan Meier survival curve for overall patients.

(a) The results of PFS curve. (b) The results of OS curve; PFS, progression-free survival; OS, overall survival.

(a)

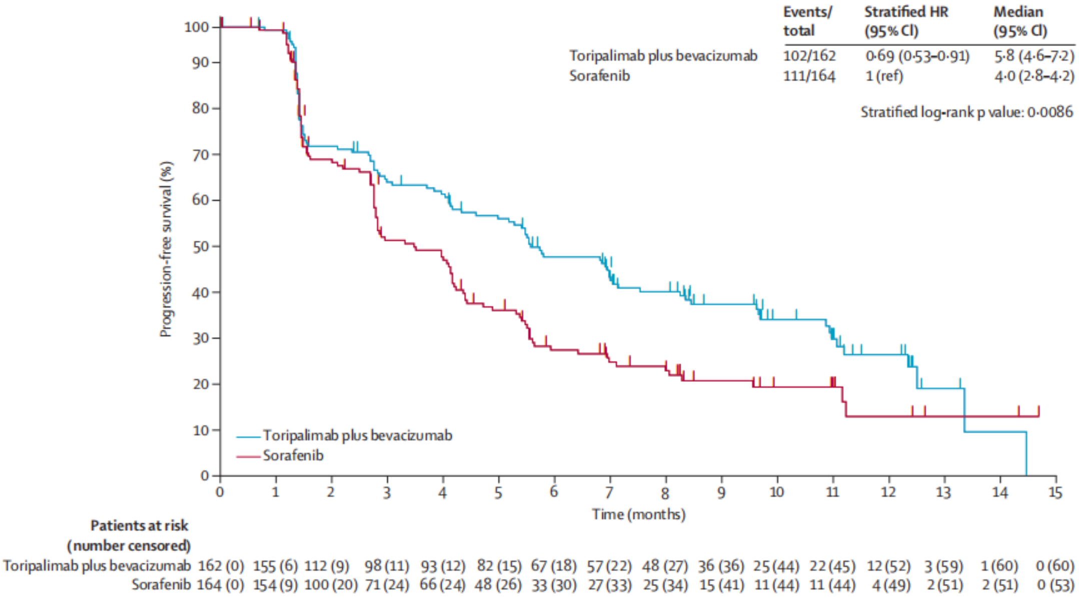

Events/ total
Stratified HR (95% CI)
Median (95% CI)
Toripalimab plus bevacizumab 102/162 0·69 (0·53–0·91) 5·8 (4·6–7·2)
Sorafenib 111/164 1 (ref) 4·0 (2·8–4·2)
Stratified log-rank p value: 0·0086
Progression-free survival (%)
Toripalimab plus bevacizumab
Sorafenib
Time (months)
Patients at risk (number censored)
Toripalimab plus bevacizumab 162 (0) 155 (6) 112 (9) 98 (11) 93 (12) 82 (15) 67 (18) 57 (22) 48 (27) 36 (36) 25 (44) 22 (45) 12 (52) 3 (59) 1 (60) 0 (60)
Sorafenib 164 (0) 154 (9) 100 (20) 71 (24) 66 (24) 48 (26) 33 (30) 27 (33) 25 (34) 15 (41) 11 (44) 11 (44) 4 (49) 2 (51) 2 (51) 0 (53)

(b)

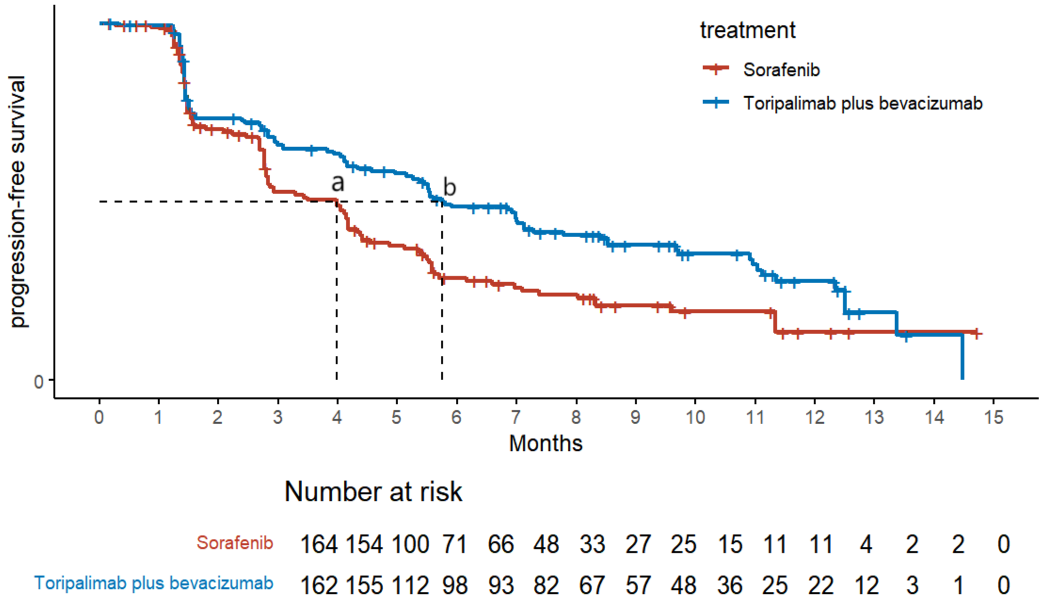

treatment
Sorafenib
Toripalimab plus bevacizumab
progression-free survival
a
b
Months
Number at risk
Sorafenib 164 154 100 71 66 48 33 27 25 15 11 11 4 2 2 0
Toripalimab plus bevacizumab 162 155 112 98 93 82 67 57 48 36 25 22 12 3 1 0

(c)

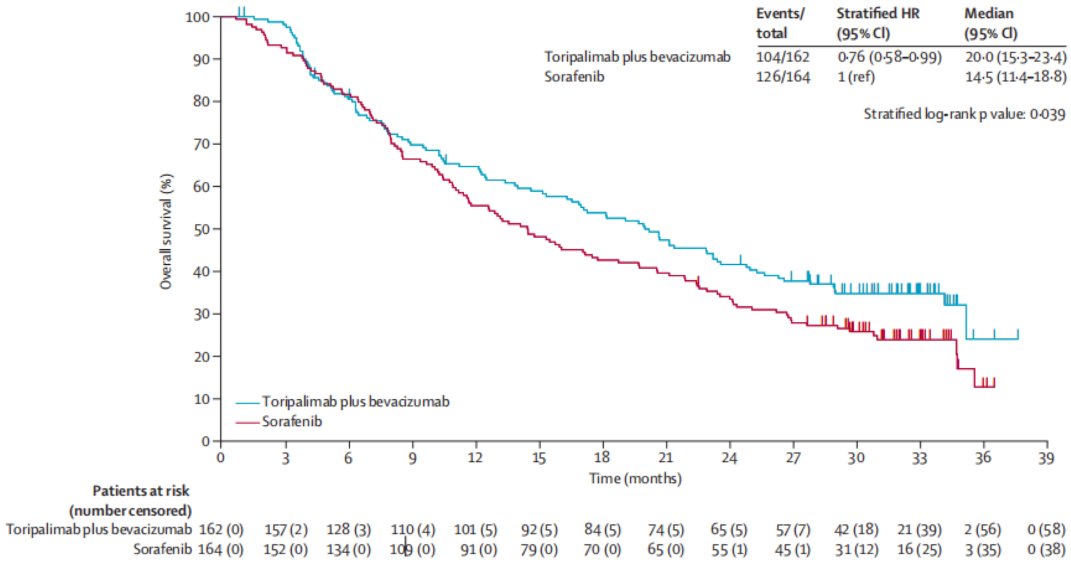

(d)

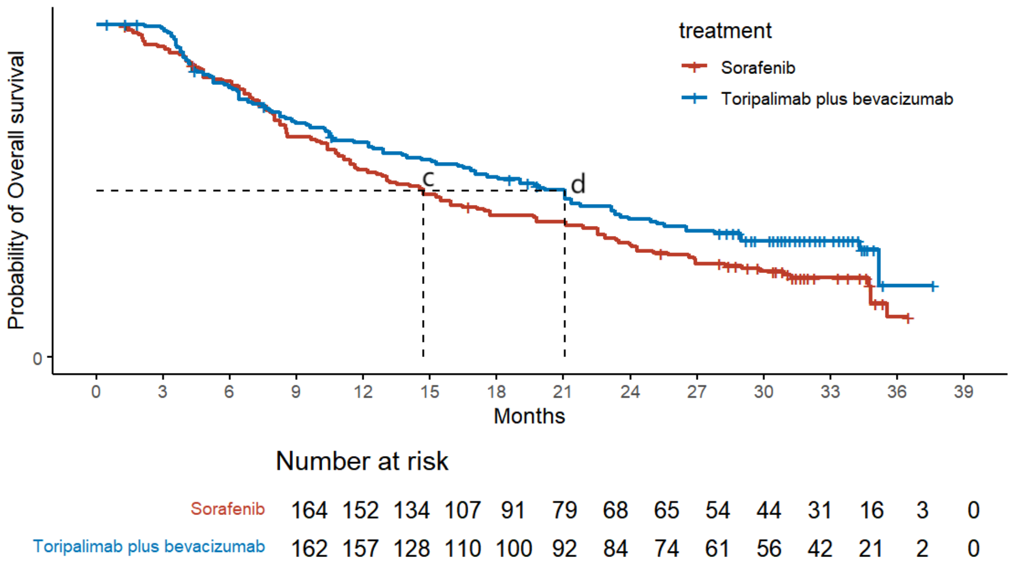

Supplementary Figure 2. Kaplan–Meier analysis of progression-free and overall survival survival.

(a) Kaplan－Meier analysis of progression-free survival of HEPATORCH.

(b) Kaplan－Meier analysis of progression-free survival of reconstruction.

(c) Kaplan–Meier analysis of overall survival of HEPATORCH.

(d) Kaplan–Meier analysis of overall survival of reconstruction.

a:Median progression-free survival of sorafenib.

b:Median progression-free survival of toripalimab plus bevacizumab.

c:Median overall survival of sorafenib.

d:Median overall survival of toripalimab plus bevacizumab.

Supplementary Table 2. HR Comparison: HEPATORCH vs. Reconstruction

**curve**	**type**	**HR**	**95% CI**
OS curve	HEPATORCH	0.76	(0.58-0.99)
	reconstruction	0.77	(0.59-1.00)
PFS curve	HEPATORCH	0.69	(0.53-0.91)
	reconstruction	0.67	(0.51-0.88)
